# The Italian version of the Pediatric Quality of Life Inventory™ (PEDSQL™) 3.0 healthcare satisfaction hematology/oncology module: reliability and validity in radiation oncology

**DOI:** 10.1186/s12955-023-02149-3

**Published:** 2023-07-12

**Authors:** Elisa Marconi, Francesco Beghella Bartoli, Elisa Meldolesi, Silvia Mariani, Giulia Panza, Alessia Nardangeli, Loredana Dinapoli, Teresa Carmen Lees, Antonella Guido, Angela Mastronuzzi, Antonio Ruggiero, Maria Antonietta Gambacorta, Vincenzo Valentini, Mario Balducci, Daniela Pia Rosaria Chieffo, Silvia Chiesa

**Affiliations:** 1 Dipartimento Diagnostica per Immagini, Radioterapia Oncologica ed Ematologia, UOC di Radioterapia Oncologica, Fondazione Policlinico Universitario Agostino Gemelli IRCCS, Rome, Italy; 2 UOS di Psicologia Clinica, Fondazione Policlinico Universitario Agostino Gemelli IRCCS, Rome, Italy; 3 Radioterapia Oncologica, IRCCS Regina Elena National Cancer Institute, Rome, Italy; 4 Great Ormond Street Hospital for Children NHS Foundation Trust, London, UK; 5 UOC di Oncologia Pediatrica, Dipartimento della Salute della Donna, del Bambino e di Sanità Pubblica, Fondazione Policlinico Universitario Agostino Gemelli IRCCS, Rome, Italy; 6 Dipartimento Oncoematologia, Terapia Cellulare, Terapie Geniche e Trapianto Emopoietico, Ospedale Pediatrico Bambino Gesù IRCSS, Rome, Italy; 7 Università̀ Cattolica del Sacro Cuore, Rome, Italy

**Keywords:** Pediatric Quality of Life, Health-related quality of life, Healthcare satisfaction, Psychometric properties, Translation, Pediatric Radiotherapy

## Abstract

**Background:**

Health-related quality of life (HRQOL) measurement has become an important health care outcome even in oncological pediatric scenario. During radiation therapy care path, pediatric patients and their relatives may suffer from emotional and psychosocial distress not only related to cancer diagnosis, but also due to the procedure and the required daily routine. Despite the high prevalence of psychosocial consequences in this setting, instruments that inquire pediatric HRQOL and healthcare satisfaction have rarely been studied in Italy. Purpose of this study was to investigate reliability and linguistic validation of the PedsQL™ healthcare satisfaction Hematology/Oncology module from its original English version to Italian language.

**Methods:**

Three phases standard procedure of cross-culture adaptation were used to create Italian version of PedsQL™ healthcare satisfaction Hematology/Oncology module. Forward translations and backward translations were performed. Finally, a pilot-testing for understandability of the ‘pre-final’ version was conducted with parents of children attending our Radiotherapy Center using two methodologies of Cognitive Interviewing (“Think-aloud Interviews” and “Respondent Debriefing”), in order to obtain the final Italian version of the PedsQL™ healthcare satisfaction Hematology/Oncology module.

**Results:**

Twenty-five parents (2 father, 23 mothers) were recruited during their children’s radiotherapy treatment and the grammatically and conceptually acceptable pre-final version of the PedsQL**™** Healthcare Satisfaction Hematology/Oncology Module was administered. The questionnaire was well understood reflecting its linguistic adaptation. Compliance with questionnaire administration was optimal. All subjects stated that the questions were interesting to express their opinion, most of them reported that all the questions of each section were clearly comprehensible and easy to understand, suggesting minimal changes that were double-checked with back translation. Furthermore, six of them spontaneously asked to complete the questionnaire in order to review the assistance received during radiotherapy.

**Conclusion:**

Our Italian version of the PedsQL™ 3.0 Healthcare Satisfaction Hematology/Oncology Module seems to be a valid and functional instrument to indagate Healthcare Satisfaction.

## Background

Measuring health-related quality of life (HRQOL) has become an important healthcare outcome in clinical practice and clinical trials. Healthcare satisfaction is now an accepted indicator of quality of care and is one of the most effective ways to assess HRQOL [1–7].

According to the WHO definition, HRQOL is a multidimensional construct that includes the domains of physical functioning (health and functional status) and psychosocial functioning (emotional, social and role functioning) [8].

Medical interest in HRQOL has also gradually emerged in pediatric oncology patients and their parents due to the potential acute and long-term side effects reported during and after oncological treatments, as well as the increase in survival [7, 9–11].

Furthermore, the literature on cancer survivors documents numerous and frequent effects on physical, psychological and relational aspects that may be related to either the disease or to the treatments [12–16].

Radiotherapy (RT), with or without chemotherapy, is still a relevant treatment option for pediatric cancer [17–21]. It consists in daily sessions repeated over days or weeks inside a treatment course with a variable duration depending on either the curative or the palliative intent of the treatment itself.

During the course of radiation therapy, pediatric patients and their relatives may experience from emotional and psychosocial distress not only related to the cancer diagnosis, but also due to the procedure and the required daily routine [22–25].

Despite the high prevalence of psychosocial consequences of pediatric cancer in patients and their relatives, instruments assessing pediatric HRQOL and healthcare satisfaction have rarely been studied in Italy.

Assessment of pediatric healthcare satisfaction could improve standards of care and help clinicians to identify unexpressed needs and to monitor outcomes in pediatric populations and their families. For this reason, it’s necessary to have a practical and validated tool to assess HRQOL.

The Pediatric Quality of Life Inventory (PedsQL) Measurement Model is a basic generic tool designed by James W. Varni, with specific modules for different pathologies that integrate the benefits of both generic and disease specific measures. PedsQL is a modular instrument that includes both self- and proxy-reports and is designed to investigate not only the biomedical endpoints, such as response rate and survival, but also to focus on behavioral and emotional problems in order to capture the daily health-related problems faced by pediatric cancer patients [26–29]. This instrument targets not only chronic health conditions but has been modified to cover also other pediatric cancer health care [11]. In particular, the PedsQL™ 3.0 Healthcare Satisfaction Hematology/Oncology Module is a widely used instrument designed to measure parents’ satisfaction of the healthcare for their children with neoplasm or hematologic diseases.

Purpose of our study was to investigate reliability and linguistic validation of the PedsQL™ 3.0 Healthcare Satisfaction Hematology/Oncology Module from its original English version to Italian language in a sample of parents with children experiencing the care-path in radiation therapy Unit in order to build an Italian version of this instrument, semantically and culturally equivalent to the original.

## Materials and methods

### Questionnaire

Italian version of the PedsQL™ 3.0 Healthcare Satisfaction Hematology/Oncology module was developed following the linguistic validation method of the PedsQL [30].

The questionnaire was developed as a parent-reported instrument to measure parental satisfaction with the healthcare for their children with hematological or oncological diseases. It consists in 25 items scale grouped into 6 domains: General Satisfaction (3 items) Information (5 items), Inclusion of Family (4 items), Communication (5 items), Technical Skills (4 items), Emotional Needs (4 items).

The questionnaire asks about how satisfied parents are with the care that their children and family have received at the hospital from the healthcare professional staff.

A 5-point Likert responses scale is used for each item (1 = very dissatisfied, 2 = dissatisfied, 3 = neither satisfied nor dissatisfied, 4 = satisfied, 5 = very satisfied).

Three phases made up the language translation process: forward translation, backward translation and field test.

A Multi-professional Group (MPG) was defined in order to proceed to the translation and testing validation of the Italian questionnaire.

The MPG was composed by 1 pediatric pyscho-oncologist, 2 radiation oncologists, 1 radiation oncologist resident and 1 English-Italian pediatric nurse.

#### Phase 1: forward translation step

Forward translation is the first step in the process of translating a questionnaire into a foreign language. It consisted in producing two different versions of the original US English instrument into Italian, with each of the translators independently producing a forward translation of the original items, instructions, and response options. (Forward translation A and Forward translation B).

Then a ‘reconciliation’ version (version n°1) was produced from the two new versions where both translators discussed the translation and agreed on a single reconciliation version.

Before starting translation, a systematic reading of the entire questionnaire has been performed by our MPG to figure out how every single item could be related to parent’s experience in our department considering our environment and the different clinical and treatment phases that configure the care path of pediatric cancer patients.

Afterwards, the questionnaire’ six dimensions (general satisfaction, information, inclusion of family, communication, technical skills and emotional needs) have been individually analyzed by our translators, to anticipate possible translation issue. In order to facilitate the understanding of the text and the administration of the questionnaire, a simple and common language was used, easily understood by all social backgrounds.

#### Phase 2: backward translation step

The second step was the backward translation of the Italian reconciliation version (Version n°1) into US English. The purpose of this process is to guarantee the accuracy and equivalence of the translated version, ensuring that the translated version (Version n°1) accurately reflects the original meaning and intent of the questionnaire items, by comparing the backward translated version and the original source version. Version 2 results from this comparison.

The process was implemented by a radiation oncologist who had no access to the original US English version of the questionnaire.

The entire process of translation was reported step by step to the Author teams by e-mail and then the project manager had been authorized to develop the Italian version of PedsQL™ (Version n°2).

#### Phase 3: patient testing

The Version n°2 of the Italian questionnaire was pilot tested on a group of subjects at the Department of Radiation Oncology, Fondazione Policlinico Universitario A. Gemelli, Rome, Italy, to determine whether the translation (instructions, items and response options) was acceptable, whether it was understood as intended, and whether the language used was simple and appropriate.

The comprehension of the questionnaire was verified with PedsQL™ Cognitive Interviewing Methodology. The cognitive interviewing consists in two modalities of interview: “Think aloud Interviews” and “Respondent Debriefing”)

First one in administer the questionnaire by reading the questionnaire out loud to the respondent “*word for word”* and give them time to think about the item.

Interviewers should not use their own words to answer a participant’s questions but should instead answer the questions by referring to the appropriate text in the questionnaire. The interviewer should provide positive comments (“Keep thinking aloud. Your feedback is excellent.”) and prompts (“Continue to tell me what you are thinking about”) not leading the respondent by suggesting agreement or disagreement with their responses.

For the second approach participant have to complete the questionnaire independently. After the respondent has completed the questionnaire, we directly ask about clarity of directions, individual items, domains, and overall evaluation of the questionnaire taking notes with each individual item.

The aim of the cognitive interviewing is to study and better understand the mental process of answering questionnaires in order to construct, create and ask better questions.

As already mentioned, both the “thought aloud” and the “through questioning of the respondent” interview techniques, were used.

During the entire process, cognitive interviews were recorded and transcribed, and notes were taken by one of the researchers (AN). By combining of the written notes together with the texts of the transcripts, an “item-by-item” summary of each section of the questionnaire and the resulting recommendations were prepared in order to confirm and validate the final Italian version (Version n° 3).

## Results

### Forward translation step

Two Italian versions of the questionnaire were produced by each translator, with no significant differences at this first reading.

A single version, the reconciliation version (version 1), was then produced by rereading each item from the two versions produced and discussing which could be the best wording to adopt in the case of discrepancies between the two translations.

Throughout the whole process of developing of the first version of the Italian questionnaire, there were no major discussions on semantic issues or major disagreements.

Below is a description, divided by dimension, of our translation issues, as paraphrased in Italian:

#### General instruction

For parent instruction we decide to translate “care” with the Italian term “Assistance” and “how happy you are with each item” with level of satisfaction to give to them the right sense in Italian.

#### General satisfaction domain

For *“The overall care your child is receiving”* item (n°1) we decided to translate the term “overall care” in the best way possible to give them the right sense of a complete assistant care.

For *“How friendly and helpful the staff is”* item (n°2) we preferred to paraphrase the meaning of this sentence besides given a literal Italian translation. We have therefore decided to use the following sentence, “The level of the reception and the support of the staff*”* which we are quoting here as a literal translation from the Italian version.

#### Information domain

To better explain the meaning of the first three items we have chosen to modify the sentences, instead of literally translating “how much information”, so we have interpreted with “Level of information that has been given”.

#### Inclusion of family domain

*For “The willingness to answer questions that you and your family may have”* item (n°2) we preferred using the Italian term of “availability” besides the “good will”, the literal translation of willingness.

#### Communication domain

For the **“***How well the staff explained your child’s disease and treatment to your child in a way that she/he could understand”* item, to give the right sense to the sentences we preferred to paraphrase the meaning of it without given a literal translation.

#### Phase 2: backward translation step

In this critical step, each translator’s primary focus was to ensure that the translated items retained the same underlying concepts as the original questionnaire. They carefully reviewed each item, analyzing its meaning and cultural implications.

The backward translated version (Version n°1) was compared with the original source version by the MPG to ensure that the final version was conceptual equivalent. (Version n°2)

Each translator focused on ensure that the translated items maintain the same underlying concepts as the original questionnaire. The backward translation process helped to identify any discrepancies in the understanding of concepts and allowed for necessary adjustments to be made to achieve conceptual equivalence.

During the backward translation process, the translators encountered two specific items, as previously mentioned in the study’s earlier section: “The willingness to answer questions that you and your family may have” and “How well the staff explained your child’s disease and treatment to your child in a way that she/he could understand.“ These items did not show perfect agreement between the two versions, as there was a slight difference due to the literal translation by the English native speaker translator during the Italian translation.

In order to resolve these minor controversies or ambiguities related to the wording, the translators engaged in thorough discussions. They delved into the meaning and intent behind the Italian version of these sentences, aiming to clarify their meaning within the questionnaire. By dissecting and scrutinizing these items, the translators swiftly arrived at a consensus on the “consensus forward/backward translation version” (Version n°2), which reflected their collective expertise and efforts.

#### Phase 3: patient testing

Between June and December 2021, 25 parents (2 father, 23 mothers) were recruited during their children’s radiotherapy treatment and the grammatically and conceptually acceptable pre-final version of the PedsQL™ Healthcare Satisfaction Hematology/Oncology Module was administered.

Parents signed informed consent prior to the interview.

All interviewees were native Italian speakers and had a child with a median age of six (range 3–6).

Compliance with questionnaire administration was optimal.

Six of the enrolled parents participated in a compilation of the questionnaire “aloud” with an interview (“think aloud method”) and the other nineteen answered the questions by self-administered questionnaire and then were interviewed by the researchers.

The overall meaning of the adapted Italian version of the module was easily understood by Italian speaker at all socio-cultural levels.

The specific items of the translated version were easily understood. The specific items of the translated version were easily understood.

The overall agreement with the questionnaire was good and there was no major misunderstanding or misleading interpretation reported from interviewees.

Regardless, we reported minimal open issue that were discussed in order to obtain our final Italian version. (Version n°3)

One interviewer, while reading the questionnaire, asked to better specify item n°4 (“How soon information was given to you about your child’s test results”) and n°5 (“How often you are updated about your child’s disease and health”) of the “Information” domain. In his opinion the questions were syntactically clear, but unrelated to their radiotherapy experience in our department, so they have been reworded in Italian like as follows: “How soon the information was given to you about your child’s clinical conditions” and “How often you are updated about your child’s clinical condition”.

Another mother pointed out to us that the item n°4 (“How much time the staff took to help you with your child coming back home”) in the “Technical Skills” domains and item n°2 (“The amount of time spent helping your child with going back to school”) in the “Emotional Needs” domains, was not relevant to her experience, also because her child was not of school age.

Therefore, we decided to make these questions more inclusive by rephrasing it as follows: “How much time the staff has devoted to helping you with your child’s dismission and/or return home” and “The amount of time spent helping your child return to school and/or everyday life”.

During this preliminary test for assessing the validity and reliability of the Italian version of the questionnaire, fifteen parents asked if they also could fill it in, as we mentioned in the previous paragraph.

Thereby we have started to collect preliminary data of parents’ health satisfaction achieving positive results.

For Information, Family’s inclusion, Communication and Technical skills’ domains the mean score was > 85, for General Satisfaction and Emotional Needs’ one the mean score was > 90.

## Discussions

We reported the results of the process of translation into and cultural adaptation to Italian of the PedsQL**™** Healthcare Satisfaction Hematology/Oncology Module.

This study aimed to develop an Italian version of the PedsQL™ 3.0 Healthcare Satisfaction Hematology/Oncology Module in order to have an instrument to investigate HRQOL and Healthcare Satisfaction and to perform psychometric assessment afterwards.

Translation process involved the cultural adaptation of the questionnaire, where semantic, idiomatic and experimental equivalences between the original version and the Italian one, were investigated.

Equivalence between the two versions were fundamental to obtain relevance and pertinence for testing the instrument in our specific setting.

To the best of our knowledge, this is the first Italian experience report the use of the PedsQL™ 3.0 Healthcare Satisfaction Hematology/Oncology Module in a sample of parents with children affected by an oncological disease.

Several studies have already evaluated reliability and validity of the transposition of different PedsQL™ Module [31–37].

Li et al. reported the feasibility and the psychometric properties of the Chinese version of the PedsQL™ 3.0 Healthcare Satisfaction Generic Module [34].

Instead, Reinfjell et al. recommended the Norwegian version of the Pediatric Quality of Life Inventory™ 4.0 (PedsQL) generic core scales for self-reports and proxy-reports for children in the age groups ranging from 13 to 15 years [36].

The absence of this kind of instrument targeting HRQOL and Heal validated for Italian, has delayed the reflection on the disease implications in this group and hampered the collection of data that could inform appropriate interventions.

For this scope, we firstly developed an adapted Italian version following the two “forward translation”, “backward translation” steps and then, through a field test, we administered the Italian version of the questionnaire to a sample population of parents with children who were experiencing radiation treatment.

During the Italian-English translation process, a few issues have arisen.

Firstly, Italian and English have different sentence structures, and directly translating sentence structures can lead to grammatically incorrect or unnatural English sentences. English language and culture have unique expressions or phrases that do not directly translate into Italian and vice versa. Cultural and linguistic differences between Italian and English can impact the accuracy of literal translations. Also, specific medical terminology in the field of hematology/oncology can vary from language to language and translators must have a solid understanding of these terms in both Italian and English.

The translators had to adapt the sentence structure, considering the cultural and linguistic norms of both languages, in order to maintain clarity and fluency, as well as being aware of the potential interpretations that could arise due to cultural differences and ensuring that the translated elements conveyed the intended meaning, as we reported in the Forward step.

Overall, the interaction between the translators during the backward translation process ensured that the Italian version of the Pediatric Quality of Life Inventory™ (PEDSQL™) 3.0 Healthcare Satisfaction Hematology/Oncology Module achieved conceptual equivalence with the original questionnaire. Their collaborative approach, combined with their careful attention to cultural, linguistic, and conceptual nuances, resulted in a final version that effectively captured the intended meaning and facilitated accurate measurement within the target population.

In particular, we focused on making the Italian version as accessible and comprehensive as possible, also taking into account social background, education and clinical conditions, by analyzing the few uncertainties highlighted in Phase 3.

Differences in education, social background, and lived experiences during the care path can significantly influence an individual’s ability to comprehend a questionnaire. These factors can affect their language proficiency, cognitive abilities, and familiarity with the healthcare system, ultimately impacting their understanding and interpretation of the questionnaire items.

To address these challenges, it is crucial to develop a questionnaire that is clear and inclusive and takes into account the diversity of the target population. This can involve using common language, avoiding specialist or technical terms, providing clear instructions, and adapting the questionnaire to different educational levels, cultural contexts and lived and perceived experience during the care pathway.

In fact, our aim was to develop a questionnaire that could cover not only the pediatric patient’s radiotherapy experience, but also their whole hospital experience, including interactions with healthcare providers and experiences in other departments and wards. This included considering their interactions with healthcare providers, nurses and other medical staff in different departments and wards. By including these aspects, the questionnaire allows for a more comprehensive assessment of the patient and family experience, enabling healthcare providers to identify areas where improvements can be made to enhance overall care and support.

The need to translate these types of questionnaires into the language of use is because measuring patient satisfaction in pediatric care could improve patient-doctor communication, increase patient and parent satisfaction, identify hidden morbidities and aid clinical decision making.

Healthcare providers can work towards providing more personalized and effective care for pediatric patients by incorporating patient perspectives and experiences into the healthcare process.

This knowledge can help identify areas where communication can be improved, leading to better understanding, trust and collaboration between patients, parents and healthcare professionals.

By understanding these hidden morbidities, healthcare providers can take appropriate steps to address them and improve the overall quality of care.

Finally, a patient-centered approach has the potential to lead to better outcomes and greater satisfaction with care. By valuing and incorporating the perspectives of patients and their families, healthcare providers can create an environment that priorities the unique needs and experiences of each individual. This holistic approach not only benefits patients and their families, but also contributes to the continuous improvement of healthcare practices and patient outcomes.

This study presents some potential limitations. Pre-test step’s sample size was relatively small and very homogeneous in terms of social class and schooling, maybe also due to the recent pandemic experience, potentially affecting the result. Moreover, for the same reason, we were precluded from applying more formal psychometric evaluations (e.g., Cronbach alpha or factor analysis) since they require a much larger population.

## Conclusion

PedsQL™ 3.0 Healthcare Satisfaction Hematology/Oncology Module is a valid and functional instrument to examine Health-related quality of life (HRQOL). It demonstrated appropriate language reliability to measure parental healthcare satisfaction in our group of pediatric cancer patients. Parent’s interest in expressing their opinion on the examined topic, might suggest the usefulness of measuring HRQOL in clinical practice and the Italian translation allows to implement these evaluations in our clinical workflow.

Preliminary results demonstrate that an assessment of parental satisfaction could provide valuable information on children’s radiotherapy pathway, helping clinicians to improve quality standards of care.

Our purpose is to continue to collect parent’s opinion during radiotherapy in pediatric patients. A prospective study could be useful to evaluate the utility of HRQOL measurement in clinical practice.

## Data Availability

Data will be made available on reasonable request due to restrictions, e.g., privacy or ethical.

## Abbreviations

HRQOL: Health-related quality of life
MPG: Multi-professional Group
